# Trauma-informed career counselling to address work traumas resulting from the COVID-19 pandemic

**DOI:** 10.4102/ajcd.v3i1.42

**Published:** 2021-09-30

**Authors:** Jennifer J. Linnekaste

**Affiliations:** 1 School of Education, Old Dominion University, Norfolk, United States of America

**Keywords:** COVID-19, work trauma, trauma-informed career counselling, narrative, Career Construction Theory

## Abstract

**Background:**

Sudden work traumas as a result of the coronavirus disease 2019 (COVID-19) pandemic have left thousands displaced from their current jobs and occupations. Traditional career counselling approaches that overlook the role of emotion are not adequate to address the numerous emotional difficulties that arise after a sudden unexpected job loss.

**Objectives:**

The objective of this research is to examine to what extent career counselling theories and interventions incorporate a trauma-informed career counselling approach and are prepared to address the emotional, psychological, and career counselling concerns that arise from work traumas as a result of the COVID-19 pandemic.

**Methods:**

A qualitative systematic literature review of all career-related journals that have trauma-informed career counselling in the title or body of the manuscript was conducted. Additionally, career related articles, books, and book chapters specifically mentioning work traumas and interventions to address these issues were also examined.

**Results:**

Only one article on trauma-informed career counselling was found given the parameters of the literature review. Currently, no articles outline how to integrate career counselling and emotion-focused therapy for trauma in order to address work traumas related to COVID-19.

**Conclusion:**

A trauma-informed career counselling approach that integrates career construction counselling and emotion-focused trauma therapy is needed to address the emotional, psychological, and career counselling concerns that arise from a work trauma as a result of the COVID-19 pandemic. An integrated approach to provide trauma-informed career counselling has been provided.

## Introduction

The global COVID-19 pandemic has had a profound impact on workers’ career trajectories and work climate resulting in unexpected job loss, significant changes within the work environment, and in some instances, traumatisation within the workplace. Globally, millions of workers became unemployed in 2020 as a result of their employer closing or losing their business because of the pandemic (BLS, 2021). Global employment losses registered 114 million; more job losses being reported in 2020 in comparison to 2019 (ILO, 2020). Those hardest hit included women (5% higher than men), younger workers (8.7% higher than older workers) and workers in the travel, food services, arts, retail and construction sectors (ILO, 2021). Unemployment and a reduction of work hours have disproportionately impacted less skilled labour harder than those in the technology sector who have been able to adapt more easily to working remotely and online. The loss of employment has been correlated with elevated depressive symptoms (Posel, Oyenubi, & Kollamparambil, 2021). In addition to the unexpected job loss, social distancing measures have forced workers to adapt to the changing work environment in unforeseen ways.

Global lockdowns and the associated restrictions have led to an increase in the number of employees working from home. Prior to the COVID-19 pandemic, approximately 17% of employees worked from home five or more days per week. But this rate has increased to 44% after the pandemic (Miltz, 2021). Workers with limited telework and technological skills suddenly found themselves striving to adapt to new software applications and programmes to retain their job. The stress of learning new skills in a short amount of time was coupled with social isolation. Workers were no longer able to socialize or discuss work-related challenges with their colleagues. Social isolation can result in psychological distress (i.e. anxiety, low mood, stress, fear, frustration and boredom) (Razai et al., 2020). Sudden adaptation to working online was seen most strikingly within the education sector, in which many teachers were forced to adapt to teaching online.

Globally, 1.6 billion students in more than 200 countries have experienced a disruption in their education because of the COVID-19 pandemic. As a result, thousands of teachers throughout the world from primary to secondary schools and universities were forced to teach remotely in order to comply with governmental measures to ensure safety during the pandemic. Whilst some schools and universities had already begun to adopt distance-learning prior to the pandemic, educators who taught solely in person expressed ambivalence about teaching remotely (Moralista & Oducado, 2020). Teachers cited concerns about academic integrity, quality of the education, student success, and the need for technological support (Moralista & Oducado, 2020). Teacher exhaustion and burnout whilst teaching online during the pandemic have been correlated with a perceived lack of accomplishment or teachers’ self-efficacy (Sokal, Trudel, & Babb, 2020). Burnout is characterised by emotional exhaustion, cynicism or depersonalisation, and low professional efficacy that can lead to anxiety, depression, and stress which in turn leads to low job satisfaction and poor job performance (Chen et al., 2020). Burnout is not only evident amongst educators, but also amongst healthcare workers which are on the front line of this pandemic.

Burnout rates for healthcare workers sharply increased worldwide as a result of the COVID-19 pandemic. In one cross-sectional survey of six hospitals in Iran, 53% of respondents (*n* = 326) experienced high levels of burnout (Jalili, Niroomand, Hadavand, Zeinali, & Fotouhi, 2021). This study mirrors a larger cross-sectional survey amongst intensive care workers across 85 countries, 12 regions, and 50% university-affiliated hospitals (*n* = 1001) in which the prevalence of symptoms of anxiety and depression or severe burnout was 46.5%, 30.2%, and 51%, respectively (Azoulay et al., 2020). Healthcare workers are faced with extraordinary challenges during the pandemic to care for and treat the patients whilst also trying to protect themselves from contracting this deadly virus. The most cited reasons for burnout include the working conditions of healthcare workers (i.e. long hours, shortage of personal protective equipment, lack of preparedness), fear of transmitting COVID-19 to their loved ones or becoming infected themselves, disruption of work-life balance, disruption to standard operating procedures and role changes, moral dilemmas (i.e. deciding who to treat) and stress related to relationships with their supervisors, colleagues, and patients (Chirico, Nucera, & Magnavita, 2021; Greenberg, Docherty, Gnanapragasam, & Wessely, 2020; Roslan, Yusoff, Razak, & Morgan, 2021). Studies are just beginning to surface within individual countries that indicate the increased risk for post-traumatic stress disorder, secondary traumatic stress, and burnout amongst healthcare workers (Vagni, Majorano, Giostra, & Pajardi, 2020).

Vocational psychologists and career counsellors have traditionally overlooked the role of emotion within career counselling (Hartung, 2011). Instead, they have taken a more intellectualised approach towards helping clients align their vocational goals and strivings with their abilities, personalities, values, and interests. Yet the adverse effects of the COVID-19 pandemic on the psychological and emotional well-being and the careers of their clients cannot be overlooked within the field of career counselling. Newer studies have found that normative stressful life events such as job loss are capable of causing post-traumatic stress (Robinson & Larson, 2010). Furthermore, high workloads, fear of contracting the COVID-19 virus, social isolation because of distancing measures, and the need to adapt to new working conditions have resulted in higher rates of traumatic stress (Boyraz & Legros, 2020; Shevlin et al., 2020).

## Trauma-informed care

Trauma-informed care is a term and approach articulated by the Substance Abuse and Mental Health Services Administration (SAMHA) in 2001 designed to inform physicians, educators, and other social providers on how to effectively approach, recognise, and respond sensitively to patients, students, and clients who have experienced trauma (SAMHA, 2015).

Trauma is defined broadly as abuse, neglect, loss, disaster, and other emotionally harmful experiences (2015). As a growing body of evidence demonstrates the significant impact of trauma on learning, behaviour, cognition, mental health, physical health, and educational success, professionals have recognised it important to outline a trauma-informed approach in order to ensure that educational institutions and service systems are aware of the policies or practices that could unintentionally further traumatise or induce trauma (i.e. abruptly removing a child from the home in the child welfare system, or harsh disciplinary practices in schools).

A trauma-informed approach helps organisations and social systems in the following ways: (1) realise the widespread impact of trauma exposure, (2) identify how trauma may impact patients/students/clients, families, and staff within the system, (3) respond by applying this knowledge into practice and institutional policies, and (4) prevent re-traumatisation (2015). As a result of trauma-informed approaches, educational and social institutions have increased trauma awareness, and they are able to incorporate their knowledge and skills into their organisational cultures, practices, and policies (NCTSN, 2021). The field of vocational psychology and counselling, however, is just beginning to address how to provide trauma-informed career counselling, particularly in light of the COVID-19 pandemic.

## Goals of the study

The goals of this study are to examine (1) what extent current recommendations for providing trauma-informed care are being utilised within the field of career counselling and development and (2) which career counselling theory and practice can address the emotional, psychological, and career counselling concerns that arise from work traumas as a result of the COVID-19 pandemic. The guiding (explorative) research questions are as follows: (1) To what extent is trauma-informed care being incorporated into career counselling practice? (2) What career counselling theories are best suited to provide counselling to individuals who have experienced work trauma as a result of the COVID-19 pandemic? and finally (3) How can career counselling provide an integrated trauma-informed approach towards work traumas?

## Research methodology

I conducted a brief, adapted, qualitative systematic review of the literature. Data was gathered by conducting a search on all career-related journal articles, book chapters, and books utilising Google Scholar and also the university library search engines that included the keywords ‘trauma-informed career counselling’, ‘work traumas’, or a combination of ‘trauma and career counselling’ in the title or body of the manuscript. The journals that did not have a SNIP score greater than 1, and a SJR ranking in top 50%, were excluded from consideration. In addition, articles that did not address career counselling practices were excluded.

Additionally, I searched and reviewed scholarly peer-reviewed articles, books, and book chapters that referenced work traumas, trauma and career, COVID-19 and career counselling implications, and those addressing emotion in the context of career counselling, until I reached data saturation, whereby repeating material overlapped with one another (Booth, 2016). Subsequently, the findings were integrated and synthesised.

## Rigour of the study

Rigorously reviewing the literature is important for planning and conducting future empirical studies (Synder, 2019). This review is comparative and deductive in nature. Because of the narrow definition of trauma-informed care (SASHA, 2005), the recent materialisation of the COVID-19 pandemic, and the theoretical limitations inherent in integrating current career theories with the current theories of trauma counselling, there were few articles, books, and book chapters for consideration, thereby increasing the comprehensiveness and accuracy of this review.

## Synthesis of the findings

The extensive search on trauma-informed career counselling yielded only two results, which include an article published in 2020 in Career Development Quarterly titled, ‘Toward Trauma Informed Career Counseling’ and an article published in 2019, in the Journal of Human Services titled, ‘Trauma-Informed Career Counseling: Identifying and Advocating for the Vocational Needs of Human Services Clients and Professionals’. Based on the exclusion criteria, the article in the Journal of Human Services was excluded because of the low SJR value (0.152) and ranking (22 986) of the journal (SCImago, 2021).

The findings reveal that the integration of trauma-informed care within career counselling is in its early stage, with respect to peer-reviewed publications and book chapters, as only one article, published in Career Development Quarterly in 2020, has offered general guidelines on how to provide trauma-informed career counselling to clients who have experienced adverse childhood experiences (ACE) (Powers & Duys, 2020). Adverse childhood experiences are traumatic experiences in a person’s life before the age of 18 which include experiencing violence, abuse, neglect, having a family member attempt or die by suicide, and growing up in a household where there is substance abuse, mental health problems, or instability because of parental separation (i.e. incarceration) (CDC, 2021). Adverse childhood experiences can increase the risk of poverty, unemployment, and attachment-related issues that can impact later relationships (Liu et al., 2013; Prescod & Zeligman, 2018; Zielinski, 2009). As a result, Powers and Duys (2020) recommend career counsellors to provide trauma-informed counselling by learning the science behind ACEs, toxic stress, and resilience, exploring ACE with clients, understanding how childhood trauma and toxic stress affects the biology of the brain, and by building connections with other healthcare providers who are aware of trauma-informed care (Powers & Duys, 2020).

In light of the COVID-19 pandemic, however, trauma-informed career counselling must go beyond tackling ACEs to also address work traumas that have arisen as a result of unexpected job loss, traumatic stress in the workplace, and disruptions in education and training in adulthood. Trauma-informed career counselling should also address the challenge of enabling people to integrate what they know about themselves consciously with their subconscious insights (Maree, 2020). Traumatisation in the workplace can be caused by or lead to a significant unwanted disruption with one’s career path or ability to continue working in one’s current position (Del Corso, 2015). In order to provide trauma-informed career counselling to this population, it is important to understand how trauma-related symptoms impact career decision-making and career adaptability. Furthermore, trauma-informed career counselling also ensures minimising the risk of re-traumatisation or replicating prior trauma dynamics by empowering clients to create a safe environment in which counsellors work collaboratively with clients to foster autonomy, control, and choice over their career decisions (SAMSHA, 2014). In order to promote resilience, career counsellors need to utilise strength-focused approaches to foster resiliency skills in their clients (2015). These protective factors include cultivating clients’ belief in their ability to cope, stay connected to their sources of support, increase their sense of self-efficacy and self-esteem, regulate their emotional arousal when confronted with problems, and create meaning to their life (Agaibi & Wilson, 2005).

After reviewing the current approaches to career and trauma counselling, I concluded that career construction counselling is well-suited for providing trauma-informed career counselling for the following reasons. Firstly, it relies heavily upon a collaborative counsellor-client relationship that affirms the client’s authority as expert and author of their life story (Savickas, 2015). This helps to foster the client’s sense of agency and autonomy over their decision-making process and reduce the likelihood that the counsellor will operate from the role of career ‘expert’ or advising consultant. An expert stance by the counsellor can replicate power dynamics felt by trauma survivors who had initially experienced a sense of powerlessness and lack of agency during the initial trauma incident (Butler, Critelli, & Rinfrette, 2011). Secondly, career construction counselling allows clients the opportunity to share their storied experience and engage in meaning making as they construct their careers (McMahon & Watson, 2008; Powers & Duys, 2020; Savickas 2020). Understanding a client’s subjective experience in narrative form allows clients the opportunity to process and make sense of their experience. For example, Trauma-focused cognitive behavioural therapy (TF-CBT) and Emotion-Focused Therapy for Trauma (EFTT), both encourage clients to share their narrative as part of the therapeutic process (Angus & Greenberg, 2011; Mannarino, Cohen, & Deblinger, 2014). As a result, narrative career interventions can be both trauma-informed, as well as trauma-focused, enabling career counsellors to participate in the primary goal of trauma recovery, in accordance with the trauma-informed care guidelines. Finally, narrative career interventions are inherently strength-based which aligns with trauma-informed care initiatives to encourage practitioners and educators to use strength-focused perspectives to promote resilience (SAMSHA, 2014). Career construction counsellors listen for stories of resiliency to empower and strengthen clients’ sense of self-esteem and self-efficacy (Pordelan, Hosseinian, & Lashaki, 2021). They help clients narrate their vocational identity and provide guiding principles to help them construct careers to overcome pain, struggles and crisis points along their career path (Savickas, 2011).

## Career construction counselling for work traumas

The COVID-19 pandemic challenges career counsellors to be cognizant and empathetic towards the emotional changes and cognitions that impact career decision making and adaptability as a result of potential work traumas. Work traumas are unanticipated painful events that cause significant emotional distress or traumatisation and result in two or more of the following: (1) Significant unwanted disruption of one’s career path and/or ability to continue working within one’s current position; (2) Re-examination of previously held beliefs about the workplace or occupation; (3) Re-examination of continuity within one’s current profession or career trajectory; (4) Vocational identity or role confusion; and (5) Erosion of one’s sense of self-efficacy and confidence as it pertains to work performance or career adaptability (Del Corso, 2015). The findings reveal that there is a lack of information on how to provide career construction counselling and trauma counselling in an integrated manner.

Career counsellors can provide career counselling to clients who have experienced a work trauma by integrating career construction counselling with EFTT because both rely upon narrative processes within the context of an empathetic, trusting, and caring counsellor-client relationship. Career construction counselling is a process by which career counsellors invite clients to share their current career circumstances or concerns in story-form. Language provides the vehicle for which reality is constructed and experienced. By understanding how clients describe their career identity and career decisions, counsellors can help clients tell a clearer and broader (or thicker) narrative about who they are, what matters to them, and how they have adapted resiliently to challenges in the past. Every career concern is unique and specific. Counsellors listen for restrictive simplified narratives that are problem saturated (i.e. I’m a failure) and that may impact career adaptability. Career counsellors help draw out these narratives and re-author problem-saturated narratives through the process of de-construction and co-construction with the counsellor.

In a similar manner, EFTT uses narrative processes to help clients assimilate their emotions, psychological needs and beliefs into a narrative that helps them organise the emotional events they have experienced (Angus & Greenberg, 2011). Many trauma narratives are incoherent because traumatic events or stressors have a disorganised influence on the self. An individual’s narrative or imagined view of oneself and the future can become disorganised and disrupted. As a result, there is a clash between two competing plotlines: the previously dominant expected of hoped for plotline and the emerging or actual plotline (Angus & Greenberg, 2011). Painful emotions may make it difficult for clients to construct a complete and emotionally coherent understanding of what they feel or think. Maladaptive secondary emotions (i.e. shame, guilt), in particular, can lead clients to avoid talking about their experiences (Angus & Greenberg, 2011). As a result, traumatic symptoms such as avoidance, intrusive thoughts, over-arousal, depression, anxiety, hopelessness, and depersonalisation can occur when clients fail to share what happened (external narrative sequence), process emotions (internal narrative sequence), and make sense of what happened (reflexive narrative sequence), (Angus, Levitt, & Hardtke, 1999; Crespo & Fernández-Lansac, 2016). Thus, the findings reveal that career counsellors can utilise the process of career construction counselling (eliciting narratives, de-construction, and reconstruction) to address work traumas that are associated with the COVID-19 pandemic, by integrating the narrative processes in EFTT (practice) with the use of narrative in career construction counselling, and in a manner that is consistent with trauma-informed care principles (SAMSHA, 2014).

## Recommendation for an integrated approach

Recommendations based upon the absence of an integrated counselling approach include: (1) eliciting the client’s narrative related to the work trauma, problem-saturated narrative impacting career adaptability, and vocational identity, (2) de-constructing problem-saturated narratives, and (3) reconstructing narratives through the process of co-construction in order to facilitate agency and adaptability. The counselling process begins by establishing safety and trust with the client. Next, career counsellors seek to draw out client narratives that relate to their present career problem, specifically, what career difficulty has led them to seek out career counselling. Depending on the type of career concern, career construction counsellors may elicit narratives that pertain to a client’s vocational identity, that is, what they consider to be their abilities, needs, values, and interests (Savickas, 2005). They may also elicit narratives that relate to their career adaptability, that is, how they have narrated their attempts to adapt or handle their current career problem. In addition, counsellors listen for signs of a break in the narrative that reflect evidence of an unprocessed work trauma. The final step is to de-construct and reconstruct/reauthor client narratives to help them have a cohesive understanding of their vocational identity and strivings to help facilitate their ability to adapt. The process of de-construction and re-authoring often happens simultaneously in the counselling process as the counsellor asks open-ended questions from a curious investigative stance, that then allow the client to begin to re-author unique stories and outcomes. As counsellors summarise and reflect back to clients’ life themes, and/or stories of resiliency within their narratives, clients are able to re-author the dominant narratives through which they perceive themselves and the world. Furthermore, the role of emotion is not overlooked. The counselling relationship serves as an emotionally corrective experience to clients as they are allowed space to narrate and make sense of their emotions which facilitate emotional regulation and adaptive coping, which in turn, help clients successfully adapt to the career challenges they face (Greenberg, 2004).

### Establishing safety

The first step in the process for providing trauma-informed career counselling is to create a safe space for clients to share their story. The relationship between client and counsellor is important in career construction counselling because it serves as a secure base for which clients feel comfortable in communicating their ideas and life stories (Savickas, 2011). Trauma impacts a client’s sense of safety and trust. Therefore, establishing it at the onset of the career counselling process is vital (Rosenbloom, Pratt, & Pearlman, 1995). This can be done in several ways that include clarifying the career counsellor’s role and expectations for counselling, grounding and mindfulness techniques, and demonstrating unconditional positive regard and empathy towards the client.

Firstly, career counsellors can establish a sense of safety by clearly defining the counsellor’s role in the career counselling process so that ambiguity and confusion are minimised. Clients who seek career counselling have different expectations about counselling than those who seek personal counselling (Lewis, 2001). They may expect the counsellor to be an expert or provide them with career advice. Therefore, it is important to explain to the clients that they are the experts of their own lives and that the role of the counsellor is to provide a space and reflective audience to help the client hear their own voice, strivings, and strategies to adapt (Savickas, 2011). Open-ended questions such as ‘How can I be helpful? or how may I be useful today?’ set the stage for this collaborative relationship in order to formulate clear goals for counselling (Savickas, 2020).

Just as the client can voice what they wish to gain from the career counselling process, counsellors also have a responsibility to clarify what they can and cannot do. For example, career counsellors do not give career advice, nor can they promise to resolve the client’s immediate existing problem if it goes outside the scope of career counselling. Some traumatic experiences in the workplace, for example, may require the intervention of a trained trauma specialist if the counsellor recognises signs of significant post-traumatic stress (i.e. intrusive thoughts, nightmares, flashbacks) that altogether supersede the client’s existing career concern. Career counsellors do not possess the necessary skills to address mental health disorders (NCDA Code of Ethics A.1.b, 2015). Career counsellors can, however, provide trauma-informed career counselling when the scope of the primary problem is directly related to a client’s career, as they possess the professional and therapeutic skills that differs from that of a career planner, advisor, or consultant. Career counsellors can clarify that their role is to help clients adapt to unexpected work traumas that significantly disrupt their vocational identity or career path, so that they can confidently construct or design their future movements in line with their needs, interests, and goals.

Secondly, counsellors can establish a sense of safety at the beginning of the counselling session by letting clients know that they are in control of the counselling process as the expert author of their career story (Savickas, 2005). Clients who have experienced a traumatic or painful experience in the workplace are more likely to have experienced a sense of powerlessness (Caplan, 2006). Therefore, clients should be informed that they can control the pace at which they tell their story, what information they wish to share, and can stop the process at any time if they become too emotionally aroused during the session. Grounding techniques (such as deep breathing, having a glass of water, or movement) help to engage one or more of the clients’ five senses and allows them to take a moment to reduce emotional arousal that can arise from discussing painful experiences (Treleaven, 2018). Counsellors who are trained in teaching mindfulness techniques (i.e. noticing one’s breath, bodily sensations) can help clients become aware of their surroundings and body throughout the counselling process to notice when they need to take a moment to slow down their emotional reactivity. It is important that trauma-informed career counsellors understand that it is difficult for clients to think or engage in career decision making when they are over-aroused (Van der Kolk, 2006). Therefore, it is important to help clients regulate their emotional arousal in order to solve the problem. As a result, clients will feel empowered and emotionally safe.

A final way to establish safety is for the counsellors to demonstrate warmth, empathy, and hope. Many trauma survivors have experienced a diminished sense of self-esteem and self-efficacy (Allen, 1995). Research has found that individuals who have undergone a traumatic experience have lower expectations for empathy from their career counsellor. Therefore, it is important that the career counsellor demonstrate empathy and support to clients at the beginning of the counselling session in order to build trust (Coursol, Lewis, & Garrity, 2001). Conveying warmth, empathy, and compassion also creates a sense of psychological and emotional safety for clients to cope with their maladaptive feelings of shame and guilt. Additionally, it is important for counsellors to be hopeful for clients as many clients may struggle with a sense of despair or hopelessness as a result of their work trauma. Career counsellors can cultivate hope by believing in the strength-based nature of career counselling to help clients imagine a future self and career possibilities.

### Eliciting the narrative

The next step in the counselling process is for the counsellors to draw out client narratives that relate to the client’s reason for seeking career counselling. The narrative surrounding the existing career concern is an important one, as counsellors are provided a window into how the client frames their perspective of the existing problem. Career problems may involve a combination of challenges related to what to do in their current situation and how to construct a future career in line with their ideal self (Savickas, 2005). Clients may express difficulties related to career concern, control, curiosity, and/or confidence. These narratives also reveal how clients feel about their current situation. It is not uncommon for clients to be unsure as to what they need or how a career counsellor can help them. They may simply report feeling stuck or confused, but they now know exactly why is it so. Trauma, in particular, can result *in unstoried emotions* which are undifferentiated and maladaptive emotional states which are not embedded in a narrative context (Angus & Greenberg, 2011). These narratives often fail to identify a specific cause or context for an emotional response as a person may not fully have insight into what their emotions mean. As a result, clients may make statements such as ‘I don’t know why I feel this way’. In response, counsellors can use emotional and content paraphrases to demonstrate patience and empathy as they help draw out the client’s narrative and clarify their career concern.

Additionally, counsellors can ask open-ended questions or utilise prompts to help clients who express difficulty knowing where to begin. When clients have experienced an abrupt disruption in their career development, it can be helpful to ask them to begin their story by picking a starting point when life made sense or felt normal to them. This helps the client organise their narrative in such a way that the arc, or episodic plotline, can be easily traced. It also enables counsellors to easily identify where the clash between two competing plotlines (the expected, or hoped for, and the emerging, or actual, plotline) exists (Angus & Greenberg, 2011). These disruptions can be identified by listening to how a client expresses their current career problem in before and after terms (i.e. ‘Before this happened, I felt and or thought this about myself or others, but now I feel or think this’).

## Deconstructing and reauthoring work trauma narratives

After having elicited a client’s narrative, trauma-informed career counsellors can help clients make sense of their experiences by deconstructing narratives in order to co-construct new strength-based stories with the counsellor who facilitate personal agency to help clients adapt to their situation (Merscham, 2000). This is done through the simultaneous process of deconstruction and re-construction, or re-authoring through a co-constructive process with the counsellor (Savickas, 2011).

Deconstruction is the process of externalising one’s internalised discourse and opening up space in one’s story to understand how the clients make sense of themselves and their life experiences (Brott, 2017, p. 97; White & Epstein, 1990). Because narratives are fluid, rather than static, they are open to interpretation and multiple meanings (Besley, 2002). This allows for counsellors to co-construct new meanings and interpretations that can help clients move forward with respect to their career concern. This process of deconstruction within career construction counselling involves utilising the Career Construction Interview (CCI) or other qualitative career assessments, designed to identify themes related to a client’s perceived interests, abilities, skills, values and preferred work environment (Savickas, 2011). Within narrative therapy, (Epston & White, 1991), deconstruction means externalising problem-saturated narratives by examining the context in which these narratives were first internalised and constructed. Finally, within EFTT, deconstruction, although not explicitly referred to as deconstruction, involves evocation and exploration of emotion in order for the client to express core primary memories and core interpersonal themes (i.e. self-doubt, feelings of failure) (Angus & Greenberg, 2011). The theme amongst each of these approaches is the process of breaking apart and analysing client narratives pertaining respectively to their vocational identity, problem-saturated stories that impact one’s vocational identity, career adaptability and emotional awareness in order to create space for unique outcomes and an alternative storyline.

Reconstruction is the complementary process to deconstruction whereby clients change or alter how their narrative, that is, how they make sense of their experiences and themselves (identity). In Career Construction Theory and Emotional Focused Therapy for Trauma, reconstruction involves the integration of new narrative and personal meanings into pre-existing view of themselves or others or a radical reorganisation of one’s self-narrative (Angus & Greenberg, 2011; Savickas, 2012). The process of reconstruction, however, differs between these two theories. Career construction counsellors reconstruct narratives by reflecting back themes to help weave a tapestry of one overarching micronarrative to create a unifying of the self (vocational identity) plotted as the protagonist within their own life story. This process is called *co-construction* in which career counsellors form a cooperative alliance and function additional audience from which clients can co-construct a future story, or unique outcome that can then be enacted upon (McMahon, 2017, p.228). On the other hand, emotion focused therapists seek to provide an emotionally corrective experience to clients through empathetic attunement (i.e. empathy, non-judging) (Angus & Greenberg, 2011). This allows for clients to feel safe to talk about their feelings and become aware of their primary emotions. Narratives change as a result of creating a space whereby clients can slow down their emotional arousal, understand how emotions impact their structures of meaning-making, and give voice to unstoried emotions of pain. As a result, clients are reconstructing, consolidating, and assimilating coherent, new, and emotionally significant ways of viewing themselves or others.

This simultaneous process of deconstruction and re-construction occurs within the context of co-construction with the counsellor. Nevertheless, it is helpful to separate out these two processes by conceptualising the deconstruction process as creating space within narratives and reconstruction process as the integration of new narratives to help clients tell an adaptive and cohesive story that enables them to adapt. Career construction counsellors work primarily to de-construct one’s vocational identity and help clients re-author who they are by weaving together a thematic story of one’s self. However, those clients who have experienced a work trauma often share problem-saturated storylines that impede career adaptability. Therefore, it is helpful to begin the process by identifying problem-saturated narratives that reflect where a client’s view of themselves, their work situation, sense of safety, or sense of purpose has been disrupted in order to address those narratives that directly impede career adaptability.

## Identifying problem-saturated stories

Problem-saturated storylines can be identified by several ways. Firstly, these narratives often reflect confusion or loss surrounding one’s vocational identity or career trajectory. Challenges reflected in problem-saturated narratives are often related to career readiness (concern for the future), career control (a sense of control over it), career curiosity (exploration of possible selves and social opportunities) and career confidence (to design an occupational future and execute plans to realise it) (Savickas, 2020). Clients who have experienced COVID-19 related fatigue and stress as a result of their work might say, ‘I don’t know if this is where I’m meant to be any longer. I can’t keep continuing to work under these conditions’. Furthermore, clients who have experienced an unexpected obstacle within their education or a job related to the COVID-19 pandemic may say, ‘I don’t know what to do now with my life. I feel lost’.

Problem-saturated storylines often involve unstoried expressions of grief. Work traumas create significant emotional distress because of the disruption of core representation models of the self and others. As a result, clients often experience feeling immobilised, helpless, panic, fear both during the traumatic experience and in the acute phase succeeding it if the threat to safety has not been neutralised (Levers, 2012). Anger, sadness, fear, and shame can persist as clients experience a lack of control over their situation. As a result, these problem stories can be identified because they tend to be narrow, thin storylines that reflect shame, anger, or sadness that rarely reflect the complexity and numerous contexts that influence a client’s behaviour, emotions, and cognitions. Counsellors can identify these storylines, as well, by attending to where the greatest degree of emotional pain or arousal is present when the clients express their narrative.

Problem-saturated storylines can also be identified by attending to stories in which the clients often repeat over and over that reflect a contradiction between a core belief a client holds about him or herself in contrast to their behaviour, feelings, or thoughts. The inability to hold a cohesive storyline about one’s vocational identity indicates that a problem-saturated story is present. In order to adapt, clients must create a narrative that does not contradict core beliefs of themselves.

Finally, problem-saturated storylines can be identified by attending to the primary arc of the client’s storyline. McAdams (2006) identified two distinctive narrative scripts, namely redemption and contamination, that people employ when constructing their life stories and adapting to obstacles and transitions. In a redemption sequence, an individual narrates a transformation from a negative state to a positive one (McAdams, 2006). This sequence describes how an individual endures, seeks to reduce suffering, improve, or redeem her- or himself in her or his present situation. Redemptive scripts are associated with personal agency, self-efficacy, strength, and confidence (McAdams, 2006). This script is also associated with how an individual becomes the hero amidst his or her life trials. By contrast, a contamination script describes a loss of a once-positive state, one that has now become contaminated or ruined.

## Deconstruction

After the counsellor has identified key problem-saturated dominant narratives that reflect a significant disruption to how a client perceives themselves now in contrast with how they perceived themselves previously, it is important to help break apart or create space into these narratives through a process of inquiry by breaking down larger narratives into smaller ones, widening the audience, exploring the context in which these dominant narratives were first written, examining language, exploring exceptions to the current narrative, and exploring the role of emotion. Each dominant narrative that poses a career adaptability problem for clients can be broken down into smaller narratives by asking clients to elaborate, explain, or provide details related to how they have made certain conclusions about themselves and others. Later during the reconstruction process, the counsellor can reflect back values and interests that thematically run through a client’s career history, thus, clarifying the client’s guiding fiction or ideal self. This will allow them to evaluate future career decisions in light of this fuller narrative.

### Exploring and expanding the client’s audience

Another way in which career counsellors can deconstruct narratives is to widen their client’s audience. A person’s audience includes significant individuals (i.e. family, friends, teachers, work colleagues, communities, culture) who influences how clients both shape and make sense of their vocational identity and career behaviour (Del Corso & Briddick, 2015). The ‘self’ does not exist apart from the feedback of an audience. Self-making is a task (Savickas, 2011). Counsellors can widen the audience by conducting an inquiry into past audiences, present-day audiences, and different audiences who have influenced or impacted the dominant problem-saturated narrative. The goal is to help the person deconstruct how the narrative was formed within a specific historical and developmental context. Furthermore, they can begin to reflect on how individuals in their present-day audience perceive their situation. This inquiry allows clients to consider how their relationship or membership within a specific audience contributes to their emotional pain and distress. Counsellors can intentionally invite new voices from a different audience into the conversation, including that of the counsellor, for consideration by the client. The goal is to open up space within a client’s rigid thin restrictive narrative.

Expanding a client’s audience allows space to explore how this audience influences each client’s interpretation of themselves, their choices, and their current career problem. This helps clients evaluate their narrative through the lens of various perspectives in order to help clients actively choose, rather than passively internalise, the feedback they receive from others. As a result of this inquiry, clients who have experienced burnout in the workplace as a result of the COVID-19 pandemic, for example, may realise that the problem is not the type of work they are doing, but rather the audience, or employer, that they are working for. Additionally, this deconstruction process allows clients to actively consider the motivation and the intent behind the feedback from others. As clients expand their audience and critically reflect on how they have formed key constructs about their vocational identity and career behaviour, they are able to employ more adaptable strategies by repositioning themselves relative to their career problem as they recognise their own influence over their self-narrative (Savickas, 2020).

### Exploring the role of emotion

Work traumas create significant emotional distress because of the disruption of core representation models of the self and others. As a result, clients often experience feeling immobilised, helpless, panic, fear both during the traumatic experience and in the acute phase succeeding it if the threat to safety has not been neutralised (Levers, 2012). Anger, sadness, fear, and shame can persist as clients experience a lack of control over their situation. Consequently, emotion influences how narratives are written in several ways. Firstly, individuals may narrate their experience in congruence with their emotional state. As a result, they may only attend to information that is consistent with their current feelings and discount information that does not. Secondly, they may interpret the motivations of others or their situation in light of how they feel. If, for example, a client feels overlooked or discarded, they may conclude that it was an employer’s intention to make them feel this way because they (the employer) did not care. Finally, undifferentiated states of high emotional arousal can make clients unable to affectively label internal emotional states or do so within the client’s limited range of feeling words. As a result, these narratives can appear disorganised. Clients may also repeat words such as ‘hopeless’ without fully being able to label the underlying emotions of frustration, fear, and sadness. Therefore, counsellors can work to help de-construct problem-saturated narratives by using externalising problematic affective states to help separate the client from the problem (Merscham, 2000). When clients can externalise negative emotional states that impede adaptability by naming the problem (i.e. ‘the anger’, ‘the depression’, ‘the injustice’), it increases a client’s sense of agency by emplotting themselves as the dominant protagonist in their life story. It also helps clients facilitate reflexivity and differentiate from their emotional state by recognising that the nature of emotion is fluid and temporal.

### Exception questions

A final way to de-construct problem-saturated narratives is to inquire into areas of exceptions to when the problem is not occurring or present, because these exceptions are entry points into alternative stories (Gonçalves, Matos, & Santos, 2009). Trauma-informed career counsellors utilise strength-focused approaches to foster resiliency skills in their clients (SAMSHA, 2014). Exception questions are predominantly used with solution-focused therapy to help clients consider alternative stories or experiences that run counter to their narrative of the existing problem. This allows counsellors to create additional space within these problem-saturated narratives to consider an alternative story or narration of their experience.

Contradictory statements within the clients’ disorganised narratives are a helpful starting point for exploring exceptions to dominant storylines that impede adaptability. The counsellor can ask about times when the existing career problem is not present. Are there times when the client does feel hopeful, trusting, or more confident? If so, what is different about those times? In addition, counsellors can ask clients who are struggling with hopelessness or defeat, exception questions related to times where they have experienced something impossible turning out to become possible, or a time when something turned out better than they expected. These types of hope-focused exception questions can instill hope and allow clients reflexively think about times, experiences, and stories within their past that have been overlooked (Larsen, Edey, & Lemay, 2007). Exception questions enable clients to articulate experiences and feedback from others that contradict his dominant problem-saturated story line, which in turn facilitates re-authoring new possible meanings and outcomes to facilitate successful adaptation.

## Restructuring and reauthoring through co-construction

As stated earlier, the complementary process to deconstruction is reconstructing or re-authoring client narratives through the process of *co-construction* with a counsellor. As counsellors deconstruct client narratives, they also seek to co-construct and re-author client narratives to facilitate adaptability by reflecting back to the client a new or expanded narrative to help the client gain an alternative awareness or meaning. Whilst the process of deconstruction naturally leads to re-construction as the clients explore how they have made sense of themselves and their experiences, counsellors intentionally participate in the construction and re-authoring process in several ways.

Firstly, counsellors help clients organise their lived experiences into a larger unfolding life story, thus, participating in the client’s self-organising process. Individuals who have experienced a work trauma often have difficulty accommodating these stories into their larger career story because of the disruptive nature of trauma (Reisberg & Hertel, 2004). Therefore, career counsellors can help clients create a storied understanding of what happened, what was felt, and what it means (Greenberg & Agnus, 2011). This enables clients to cognitively process, organise, structure, and ultimately assimilate both their emotional experiences and work trauma into their unfolding career narrative (Greenberg & Agnus, 2011).

During deconstruction, macronarratives are broken down into micronarratives and the language a client uses is elucidated and elaborated upon to provide a deeper understanding of how a client constructs and makes sense of their experience. During the reconstruction process, however, the counsellor helps weave together these micronarratives by intentionally reflecting back career themes related to a client’s interests, behaviour, career decision-making, motivation, skills, abilities, values, and needs across their vocational lifespan which can, in turn, help the client tell a unifying story about who they are (vocational identity), who they wish to be (future idea self-narrative) and how they seek to adapt (unique story outcomes) (Hartung & Santilli, 2016, p. 183; Savickas, 2011).This is often done in career construction counselling by utilising the CCI, whereby clients are asked to reflect on their role models, hobbies, favourite and least favourite school subjects, favourite television shows, books, stories, and magazines or websites that interest them (Savickas, 2011). As clients answer these questions, themes appear. As these themes are reflected back to the client, it helps them organise their experience.

Secondly, counsellors help clients reconstruct narratives by not only intentionally inquiring about past and present audiences that impact the client’s current career concern but also as participating actively as another audience who helps a client tell the story about who they are and where they are going. The relationship between the counsellor and client is at the heart of constructivist career counselling as well as emotion-focused narrative therapy. Constructing a sense of self involves an ongoing process both of identifying with and symbolising emotions and actions in a way that provides temporal stability and coherence (Angus & Greenberg, 2011). This is not done in isolation but within the context of a warm, empathetic, relationship in which a highly attuned therapist attempts to focus the client’s attention on emotions and experiences outside of their client’s awareness in order to offer meaning that will help the client clarify and co-construct new meanings (Angus & Greenberg, 2011). By assimilating primary emotions, such as sadness, clients can re-organise and re-author a dominant contamination script of loss into a redemptive script of growth and development.

Thirdly, counsellors help clients identify adaptive schemas and strategies where clients have used in the past or wish to use to in the future to overcome career adaptability challenges through the use of exception questions as well as expanding one’s audience to consider role models or fictional characters that have overcome similar hardships. Clients are seen as change agents functioning as authors and actors within their life story who possess knowledge as to how to best adapt and adjust to their situation as experts of their own lives (Bruner, 2004). As such, career construction counsellors believe that clients tell themselves the stories they need to hear (Savickas, 2020). Clients may not be able to make the connection between these stories and how they relate to their career concern as they often lie on the periphery of their consciousness. Furthermore, for clients who have experienced a work trauma, it may be even more difficult for them to hear the advice and encouragement they are telling themselves because of emotion, anxiety, or disorganised thought patterns. Therefore, counsellors are cognizant and mindful to use the client’s words and systems of sense-making whilst they reflect or ask questions to help clients hear their own voice (Bruner, 2004). There are several ways to identify adaptive schemas. Counsellors can ask clients to share a motto they live by, books or movies that resonate with them, or times in the past where they felt contrary to how they feel today. Mottos, metaphors, and key stories that a client shares often reflect the advice a client has for themselves with respect to the work trauma they currently face.

Lastly, counsellors can help facilitate narrative change by presenting alternative storylines, meanings, and reflections that the clients may then choose to incorporate into their own autobiographical narrative. The goal is to reconstruct a shift within a client’s perception that prompts a new understanding by which they can construct an action plan that will enable them to make career decisions (Savickas, 2020). For example, a counsellor might reflect back that they hear the story of a worker who has faced incredible challenges only to continue striving and who has not given up. In turn, the clients may choose to internalise the counsellor’s perception, thereby co-constructing a new narrative that enables them re-author problem-saturated narratives that impede adaptability.

## Limitations of the study

The review of current trauma-informed career counselling research is limited by the degree to which articles appeared in the selected search engines and university databases. The term trauma-informed career counselling is relatively new and therefore, relevant materials related to trauma and career counseling may have been excluded. This qualitative overview and recommendation for an integrated model is consistent with the author’s area of expertise and knowledge in the area of Career construction counselling and may overlook efforts made within the field of trauma counselling or psychotherapy or social work to address work-related issues related to trauma. The integration of EFTT was selected because of overlap in utilising client narratives to promote growth and healing. However, this can result in excluding other trauma therapies such as TF-CBT that may be integrated with alternative career theories such as Happenstance and Social Learning theories of Career Counselling.

## Implications for research, practice, policy, and future research

There is a need for future quantitative studies to be conducted to test the efficacy of the integrated approach outlined in this article. In addition, empirical studies are needed to compare traditional with trauma-informed career counselling approaches in order to evaluate outcomes in comparison to the trauma-informed career goals (i.e. promoting strength, resiliency).

## Conclusion

Few career counselling and development theories have integrated trauma-informed care into practice and provide a conceptual basis for addressing the emotional, psychological and career counselling concerns that arise from work traumas experienced as a result of the COVID-19 pandemic. Counsellors can provide trauma-informed career counselling to clients who have experienced a work trauma as a result of the COVID-19 pandemic by integrating career construction counselling with EFTT. Both CCT and EFTT rely upon narrative processes that utilise strength-based, resilient approaches to help clients emotionally cope and adapt to problem-saturated narratives that impact career adaptability. This article explains how career counsellors can utilise the process of co-construction (eliciting narratives, de-construction, and reconstruction) to address work traumas that are associated with the COVID-19 pandemic in a manner that is consistent with trauma-informed care principles (SAMSHA, 2014). Trauma-informed career counsellors can help clients make sense of their experiences by deconstructing narratives in order to co-construct with the counsellor new strength-based stories that facilitate personal agency for the clients (Merscham, 2000). In conclusion, the field of career counselling and vocational psychology need to develop theories and trauma-informed career interventions to address trauma in the workplace.
